# The Reward-Related Shift of Emotional Contagion from the Observer’s Perspective Correlates to Their Intimacy with the Expresser

**DOI:** 10.3390/bs13110934

**Published:** 2023-11-15

**Authors:** Ying Chen, Wenfeng Chen, Ling Zhang, Yanqiu Wei, Ping Hu

**Affiliations:** Department of Psychology, Renmin University of China, No. 59 of Zhongguancun Street, Haidian District, Beijing 100872, China; chenying2019@ruc.edu.cn (Y.C.); wchen@ruc.edu.cn (W.C.); ymtxlc@ruc.edu.cn (L.Z.); weiyanqiu@ruc.edu.cn (Y.W.)

**Keywords:** emotional contagion, social function, reward, late positive component (LPC), intimacy

## Abstract

Although previous studies have found a bidirectional relationship between emotional contagion and reward, there is insufficient research to prove the effect of reward on the social function of emotional contagion. To explore this issue, the current study used electroencephalography (EEG) and the interactive way in which the expresser played games to help participants obtain reward outcomes. The results demonstrated a significant correlation between changes in emotional contagion and closeness, indicating that emotional contagion has a social regulatory function. Regarding the impact of reward outcomes, the results showed that compared to the context of a loss, in the context of a win, participants’ closeness toward the expresser shifted to a more intimate level, their emotional contagion changed in a more positive direction, and the activity of the late positive component (LPC) of the event-related potentials (ERPs) changed to a greater extent. Significantly, the mediation results demonstrated the effect of reward and indicated that changes in the LPC elicited while experiencing the expressers’ emotion predicted the subsequent shifts in closeness through alterations in emotional contagion of the anger emotion in the winning context and the happy emotion in the loss context. This study provides empirical evidence regarding the social function of emotional contagion and proves for the first time that the reward context plays a role in it.

## 1. Introduction

As the 16th-century poet John Donne said, “No man is an island”. We are always in environments in which we connect with others. The motivation to develop social bonds to meet our universal need for belonging is one of the most powerful drivers of human behavior [1,2]. Previous studies have found that being congruent with others’ emotional states can lead to feelings of connectedness [3], indicating that this emotional contagion has a social regulatory function [4]. However, little attention has been paid to the boundary conditions that affect the social functioning of emotional contagion. Factors that affect social behavior may be effective owing to their rewarding nature [5]. Thus, the current study focuses on the reward context, which may provide an essential motive for the occurrence and changes in the social functions of emotional contagion.

### 1.1. The Social Function of Emotional Contagion

The way to establish intimate social bonds is usually embedded in daily interactions, especially emotional interaction. Our emotions are often spontaneously influenced by others during an interaction. For example, we may feel happy when we observe others smiling happily. Researchers have broadly defined the phenomenon of emotion transferring from expressers to observers as “emotional contagion” [6,7]. Emotional contagion is considered functional, as it facilitates interpersonal understanding, closeness, and coordination [4,8,9,10,11,12,13]. Evidence supporting the social functioning perspective has revealed an association between emotional contagion and the quality of social bonds. Kühn et al. (2011) found that irrespective of emotion, the congruent emotional state of expressers and observers leads to a higher closeness rating [13]. Other findings also suggested that the more contagious response of the observer leads to closer relationships with the expressers [4,14,15,16], and this positive consequence would also occur when expressers evaluate observers [12,17,18]. Therefore, emotional contagion and its contagious responses can serve as social regulators and promote social bonds.

### 1.2. Reward Context and Social Functions of Emotional Contagion

The positive consequences of emotional contagion may not occur in all cases, depending on the interactive context. In addition to the type of emotion, researchers have suggested that the context of interactions with outgroup members, higher-status partners, or partners who are not interested in interaction can affect the consequences of contagious responses [19]. Hess (2021) insists that only when the relationship between two interacting people is positive or when they hold a positive attitude toward each other—that is, when there is an affiliative motivation or goal for the interaction—can the contagious response fulfill its social function (leading to closer relations) [20]. This viewpoint is supported by evidence that contagious responses are more likely to manifest in contexts that invite affiliation than in antagonistic contexts [14]. Emotional contagion with unpredictable or non-affiliative members may call for higher consumption of cognitive resources [19,21]. Thus, predictable affiliation may play a crucial role in achieving better social functioning during emotional contagion. However, there is little direct evidence regarding the factors that affect the social regulatory function of emotional contagion.

Importantly, these factors may influence social behavior through their rewarding nature. Researchers have suggested that factors that regulate contagious responses such as liking (like/dislike), competition (cooperation/competition), and group membership (ingroup/outgroup) have reward properties [5,22]. Considering this rewarding nature, it can also affect expressers’ emotions when directly adding reward values to interactive expressers. This was supported by evidence that when neutral faces were previously associated with rewarding outcomes (i.e., rewarding faces), observers had a greater contagious response to the happy expressions of these faces [5,22,23]. This is in line with the social function view of contagious responses [2], which suggests that mimicking responses associated with a higher reward probability may help ensure future returns [10,24,25]. Therefore, it can be inferred that affiliative and predictable rewards may be key contextual factors for the improved functioning of social regulation in emotional contagion. However, little or no attention has been paid to this issue. This study investigated, for the first time, the impact of rewards on the social function of emotional contagion.

Previous studies have primarily manipulated rewards by forming memories or experiences of rewards (gains or losses) attached to social stimuli (faces), which can alter the reward value of faces, thereby promoting changes in response to facial emotions. However, the influence of previous experience may not necessarily be the cause of subsequent behavioral changes [26], and the association between reward and face was not related to subsequent task performance in these studies. Evidence shows that real-time rewards that appear in dynamic interactions may have different effects from rewards of previously associated memories [27] and that real-time rewards can create a reward context that is in line with the dynamic characteristics of social interaction in daily life [28]. Trilla et al. (2020) attached rewards to neutral faces through real-time selection by participants, which determined the reward outcome of the face [26]. However, their findings regarding the effectiveness of reward outcomes were influenced by memories previously associated with these faces rather than serving as a real-time context for the occurrence of a contagious response. It remains unclear whether and how the context of rewards affects emotional contagion and its promotional effect on social bonds. 

Hein et al. (2016) assigned the initiative to receive rewarding outcomes (avoiding electric shocks) to expressers rather than participants through the interactive form of an expresser group helping or not helping participants avoid electric shocks [29]. This is different from situations in which participants earn rewards by playing games on their own, as this is more likely to improve the level of reward interaction between participants and expressers and establish positive connections with expressers [29]. Inspired by their research paradigm, the current study adopts an interactive approach in which expressers play games for participants to obtain reward outcomes and explore the impact of the real-time context of the reward outcome on the social regulatory function of emotional contagion.

In addition, the neural correlates underlying the occurrence of emotional contagion are correlated with the neural mechanisms of reward processing. Previous studies have found that congruent emotional states can enhance the activities of the medial orbitofrontal and ventromedial prefrontal cortices, which are associated with reward processing [13,30]. Moreover, researchers have also indicated that the closeness brought about by interacting with others may also be supported by reward-related brain areas [31,32]. This suggests that emotional contagion and the subsequently established closer relationship are both related to brain activities during reward processing. Hence, when emotional contagion occurs in the interactive context of rewards, the reward system may support the interplay between emotional contagion and the external contextual reward stimuli, thereby promoting changes in emotional contagion and its social regulatory functions. However, the neural correlates underlying emotional contagion and their promoting effect on social relationships remain unclear. 

Electroencephalography (EEG) can elucidate the temporal characteristics of brain processing during the effect of reward outcomes on emotional contagion and its promoting effect on social bonds (e.g., closeness). Previous studies have found that the anterior brain activities of early automatic components (EAC, i.e., N1 and N2) are related to emotional arousal and emotional contagion [33]. Moreover, the late positive component (LPC) is linked to the late cognitive top-down process (such as perspective-taking and mentalizing) [33,34,35,36], which exerts top-down regulatory control over contagious responses [16,25,37,38,39]. These findings suggest that N1 or N2 may indicate the occurrence of emotional arousal and emotional contagion when experiencing others’ emotions, while LPC activity is likely to reflect the modulation of this occurrence. However, there is no direct evidence to suggest whether these activities may also play a significant role in the shift of emotional contagion and its impact on social bonds. Therefore, this study also explored and verified the EEG patterns of emotional contagion and its social regulatory functions.

### 1.3. Overview of the Present Research

Based on the literature discussed above, the main purposes of this study were to (1) examine the social function of emotional contagion in promoting interpersonal relationships (i.e., closeness) and (2) explore whether and how reward outcome affects the social function of emotional contagion.

Previous studies have found that contagious responses in an affiliative context promote interpersonal relationships to change in a more intimate direction, whereas in the non-affiliative context, the opposite is true [2,14,31]. Based on these findings, the first hypothesis of this study is that when emotional contagion changes to a more positive experience direction, the quality of interpersonal relationships (such as closeness) may also change to a more intimate direction, indicating a social promoting effect of emotional contagion. Conversely, when emotional contagion changes in the direction of more negative experience, it can predict a more distant change in closeness (H1). 

Additionally, rewards are motivating factors [40]. Motivations are what get us going and keep us moving forward; they motivate us to approach when we see a reward, whereas they drive us to hold back when we perceive a threat [41]. It has been suggested that the reward context may promote positive consequences of behaviors, such as establishing intimate relationships with others, and given the preceding discourse on neural activities, it is reasonable to anticipate that the reward outcomes can elicit alterations in neural activity while experiencing others’ emotions. Therefore, this study hypothesizes that when a reward outcome is gain, it may play a motivational role in affiliation or approach, thereby facilitating changes in interpersonal relationships characterized by increased intimacy, promoting positive shifts in emotional contagion, and eliciting heightened activity in ERPs (H2). 

Furthermore, the study explored whether reward contexts play a role in the social function of emotional contagion and hypothesized that the reward context may have a motivational effect. Specifically, when the reward outcome is gain, a more positive change in interpersonal emotional contagion is more likely to be promoted, thereby facilitating a more intimate change in closeness; that is, the social promoting effect of gain on emotional contagion. When the reward outcome is a loss, emotional contagion may change more negatively, leading to a more distant closeness; that is, the negative consequences of emotional contagion (H3). 

Finally, prior research has revealed that experiencing the emotions of others not only triggers activations in brain activity, but also elicits corresponding subjective emotional experiences within oneself [42]. This implies a potential correlation between changes in brain activity while experiencing other people’s emotions and subsequent changes in subjective emotional rating (i.e., emotional contagion). Besides, previous studies suggest that top-down processing may amplify or dampen the output of contagion and may elicit a regulated response, involving either approach or avoidance behaviors toward the emotional stimulus [43,44]. This further suggests that changes in brain activity while experiencing others’ emotions may predict subsequent shifts in emotional contagion (e.g., the subjective emotional experience on oneself) and social function of emotional contagion (e.g., closeness toward the emotional expresser), influenced by the context of reward. Thus, the current study also speculates that emotional contagion, as a core element in emotional communication, might play a mediating role in the relationship between brain activity change during the experience of others’ emotion and subsequent intimacy shift towards the emotional expresser (H4).

To test these hypotheses, this study used electroencephalography (EEG) technology and an interactive method in which expressers played games to obtain either gain or loss outcomes for participants, exploring the effect of reward context on the social functions of emotional contagion, and the underlying neural correlates.

## 2. Materials and Methods

### 2.1. Participants

Forty healthy participants were recruited for the current study, all of whom had provided written informed consent to participate in a single session and were paid an appropriate fee for their participation. All participants had normal or corrected-to-normal vision, and none had a history of psychiatric or neurological disorders. The study was approved by the ethics review committee. Two participants were excluded because of experimental procedure problems, and three subjects were excluded due to excessive noise in the EEG signal. Therefore, a total of 35 participants entered the final analysis (22 females; age: 18–28 years, *M* = 20.31 ± 3.21 standard deviation (SD)).

### 2.2. Stimuli

Four Caucasian models (two males and two females) were selected from a high-resolution 3D dynamic facial expression database developed by Yin et al. (2008) [45]. Dynamic facial expressions were chosen in this study because they are considered more ecological and, therefore, closer to daily life [46,47,48]. Two models (one male and one female) expressed happiness, and the other two expressed anger. In the current study, the emotional video clips were edited to 2000 ms in duration, changing from a neutral expression at the beginning to a full-blown emotional expression by the end. Each video clip contained a set of 40 frames per face with increasing emotional intensity. The first seven frames were neutral facial expressions, and the emotional intensity of frames 8 to 15 gradually approached the maximum emotional degree. The final 25 frames were all at the maximum emotional degree. All frames were presented for 50 ms each, with a playback speed of 80%, which created the compelling illusion that the short video clip displayed a dynamic facial expression of either anger or happiness.

Six Cyrillic letters were used in the monetary game. In each monetary game trial, two letters were presented on the left and right sides of the screen. Selecting one of the two letters resulted in a gain (win) or lose (loss) outcome. Each emotional video clip was firmly matched to the monetary outcome (either a win or a loss) of the game interaction. A further 23 participants rated the stimuli prior to the experiment (valence rating: 1–9 points, where 1 indicated very unpleasant, 5 indicated neutral, and 9 indicated very pleasant; arousing rating: 1–9 points, where 1 indicated very calm (even drowsy), and 9 indicated extreme excitement). The mean valence rating scores of the video clips were as follows: 7.13 (SE = 0.269) for the happy-win video, 6.79 (SE = 0.187) for the happy-loss video, 3.57 (SE = 0.234) for the anger-win video, and 3.52 (SE = 0.250) for the anger-loss video. There were no significant differences between happy-win and happy-loss (paired *t*-test: *t*_(22)_= 2.072, *p* = 0.051, Cohen’s d = 0.432) or between anger-win and anger-loss (paired *t*-test: *t*_(22)_= 0.188, *p* = 0.852, Cohen’s d = 0.039). The mean arousal rating scores of the video clips were 6.13 (SE = 0.276) for the happy-win, 6.00 (SE = 0.243) for the happy-loss, 5.87 (SE = 0.269) for the anger-win, and 5.78 (SE = 0.208) for the anger-loss. There was no significant difference in the arousal scores between the happy-win and happy-loss (paired *t*-test: *t*_(22)_= 0.130, *p* = 0.665, Cohen’s d = 0.092) or between anger-win and anger-loss (paired *t*-test: *t*_(22)_= 0.087, *p* = 0.628, Cohen’s d = 0.103).

### 2.3. Procedure

**Figure 1:**
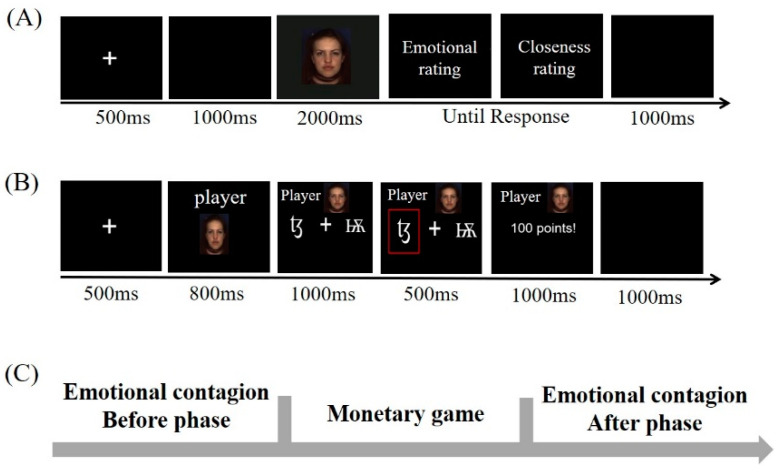
(**A**) The emotional contagion phase before and after the monetary game. (**B**) The monetary game in which the expressers play the game for participants. (**C**) The experimental progress of a trial in the experimental program.

The whole experiment consisted of three blocks. There were 64 trials in each block, and each trial was presented in a counterbalanced order. Each trial included three phases: the money game process in the middle phase and the emotional contagion process before and after the game (before and after phases), as shown in Figure 1C. In the before and after phases of each trial, as shown in Figure 1A, the participants watched an emotional video clip (happy or angry) and then scored their current emotional experience (emotional contagion process) and subjective closeness toward the expresser. The emotional video was the same in the before and after phases of a trial. In the monetary game, as shown in Figure 1B, the model in the video reappeared as a player, played the game, and finally obtained the corresponding win/loss outcome for the participants (i.e., the outcomes of the monetary game were related to the participants’ fees). The monetary game was modified by Lockwood et al. (2016) and Hein et al. (2016) [29,49]. The participants were asked to observe the game process and pay attention to the outcome (win or loss) during the monetary game. Therefore, there were two phases of emotional contagion in each trial to compare emotional changes in emotional contagion.

The before, middle, and after phases of each trial started with a “+” fixation for 500 ms and ended with a blank screen for 1000 ms. In the before and after phases of emotional contagion, an emotional video clip appeared for 2000 ms. During the playback of this emotional video clip, participants were asked to fully experience the expressers’ emotions, imagine communicating with the people in the video, and then evaluate their current emotional experience after the video clip disappeared (emotional rating: 1–9 points, where 1 indicated very unpleasant, 5 indicated neutral, and 9 indicated very pleasant). After the emotional rating, participants were asked to evaluate the closeness of their relationship with the model in the video clip (closeness rating: 1–6 points, where 6 represented not at all, and 1 represented very close). Emotional and closeness ratings were self-paced, and the screen disappeared after the participant’s response. 

In the monetary game, a pair of meaningless Cyrillic letters appeared on the left and right sides of the screen for 800 ms, and one was selected by the player (selection screen) for 1000 ms. Subsequently, the outcome (win 100 points or lose 100 points) appeared for selection. Participants were not asked to respond but were asked to carefully observe the entire monetary game process. Figure 1 illustrates the specific process of each trial.

### 2.4. Data Acquisition and Analysis

Neural activity was recorded using an EEG recording system with a 64-channel BIOSEMI Active-Two amplifier system (BioSemi, Amsterdam, the Netherlands) that automatically conducted a notch filter (https://www.biosemi.com/faq/adjust_filter.htm, accessed on 10 September 2022). The head surface was arranged according to the extended international 10/20 EEG system [50]. A scalp EEG was recorded at 2048 Hz with a 0.1 to 417 Hz bandpass filter and with the common mode sense active electrode and the driven right leg passive electrode as the reference and ground electrodes, respectively. The EEG data were preprocessed using MATLAB 2018b (MathWorks) with the EEGLAB 2020 toolbox. EEG signals were resampled offline to 512 Hz. Noisy trials were identified visually and discarded. Extreme amplitudes were rejected using a dynamic procedure with a mean threshold of ±75 ivy, ensuring an average of 80% of trials for each condition. EEG deflections resulting from eye movements, blinks, muscle artifacts and other noise were corrected using the ICA procedure [51]. 

Event-related potential (ERP) analysis was filtered using a bandpass filter ranging from 1 to 30 Hz. Offline reference is the averaged signals of all brain electrodes. All epochs were baseline-corrected using a 500 ms pre-stimulus window. The average amplitude of three adjacent electrodes for each region was calculated. For example, the midline frontal regions were electrodes Fz, F1, and F2; the left-frontal regions were electrodes F3, F5, and F7; and the right frontal regions were electrodes F4, F6, and F8. Three ERP components of interest were analyzed: N1(100–120 ms), N2(260–340 ms), and LPC (600–900 ms).

### 2.5. Statistical Analysis

To explore the impact of reward outcomes on subjective closeness, subjective emotional experience, and EEG activity (H2), we conducted a repeated-measures analysis of variance (rmANOVA) in SPSS 20.0. The independent within-subjects variables included phase (before, after), emotion (anger, happy), and outcome (win, loss). The significance level was set at *p* < 0.05 for all analyses. *p*-values in the statistical test were. The Greenhouse–Geisser correction was applied to account for sphericity violations whenever appropriate. Post hoc testing of the significant main effects was performed using Bonferroni adjustments. Partial eta-squared (*η_p_*^2^) values were calculated to indicate the effect size in the rmANOVA models, with 0.05 representing a small effect, 0.1 representing a medium effect, and 0.2 representing a large effect [52].

To examine the impact of reward outcomes on the social function of emotional contagion and the underlying mechanistic processes (H1 and H3), Pearson’s correlation coefficients were calculated to investigate the relationship between the reward-related changes in behavioral ratings (both closeness and emotional contagion) and reward-related shifts in EEG activities. Then, we also performed a mediation analysis to investigate whether the reward-related shift in brain activities when experiencing expressers’ emotions promoted the subsequent changes in emotional contagion, which in turn facilitated the later social bonding (i.e., intimacy) alterations (H4). Correlation and mediation analyses were conducted in MATLAB 2018b. For the mediation analysis, the Multilevel Mediation and Moderation (M3) toolbox [53] was employed. A total of 5000 iterations were used to test the significance of the inference statistics (the significance size was set at 0.05). The mediation analysis considered the reward-related change in EEG activity (i.e., differential activity from the before phase to the after phase) as the independent variable (X), the corresponding differential emotional experience score (i.e., the score difference in emotional experience between the before phase and the after phase) as the mediator variable (M), and the associated differential closeness score (i.e., the score difference in closeness between the two phases) as the dependent variable (Y).

## 3. Results

### 3.1. The Effect of Reward Outcome on the Closeness

To explore whether and how the monetary game interaction’s reward outcome affected the closeness change (H2), we first conducted a three-way rmANOVA with phase (before, after), emotion (anger, happy), and outcome (win, loss) as independent within-subjects variables. In the current study, elevated closeness scores were indicative of increased relational distance, whereas smaller scores denoted greater levels of intimacy. To better illustrate the evolving pattern of closeness, each closeness rating was subtracted from 6, rendering a higher calculated score indicative of greater intimacy.

**Figure 2:**
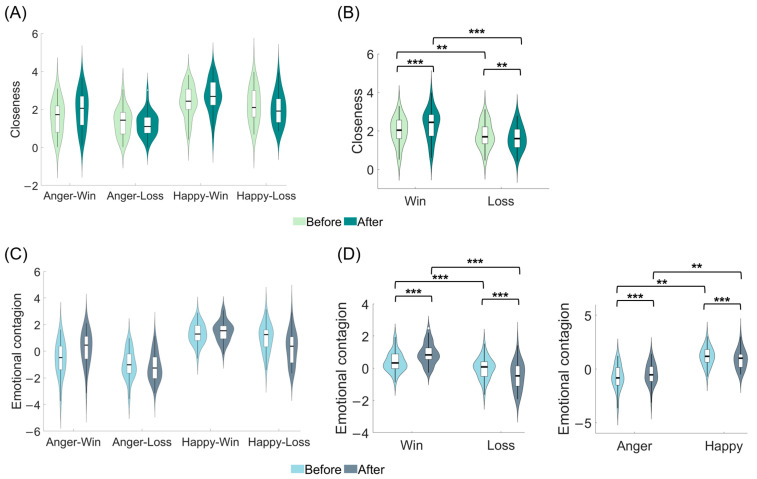
(**A**) General overview of closeness. (**B**) Closeness changed in a more intimate direction when the participant obtained a win outcome (as opposed to a loss) from the before phase to the after phase. (**C**) General overview of emotional contagion. (**D**) Emotional contagion exhibited a more positive change in response to a win outcome (as opposed to a loss), as well as when exposed to an anger expression (as opposed to a happy expression), from the before phase to the after phase. ***: *p* < 0.001; **: *p* < 0.01; error bars represent standard errors.

The results showed that the phase × outcome interaction was significant (*F* (1, 34) = 23.043, *p* < 0.001, *η_p_*^2^ = 0.404). Simple effects analysis suggested that the win condition had a significantly more intimate relationship in the after phase than in the before phase (win: after > before, *p* < 0.001), whereas the loss condition had the opposite pattern (loss: after < before, *p* = 0.005); in both the before (*p* = 0.003) and after phases (*p* < 0.001), the intimacy under the win condition was significantly greater than that under the loss condition. The main effect of phase was significant (*F* (1, 34) = 8.266, *p* = 0.007, *η_p_*^2^ = 0.196), suggesting that there was a more intimate relationship in the after phase compared to the before phase. The main effect of emotion was also significant (*F* (1, 34) = 32.408, *p* < 0.001, *η_p_*^2^ = 0.488), with the intimacy under the happy condition significantly greater than that under the anger condition. The main effect of outcome was significant (*F* (1, 34) = 33.708, *p* < 0.001, *η_p_*^2^ = 0.498), and post hoc testing revealed that the intimacy under the win condition was significantly greater than that under the loss condition. The other interactions were not significant. These results demonstrated a significant alteration in closeness, as depicted in Figure 2A,B. Accordingly, when the reward outcome was a win, participants’ subjective closeness towards the expresser shifted in a direction indicative of increased intimacy; while when it was a loss, the closeness changed in a direction suggesting decreased intimacy. 

### 3.2. The Effect of Reward Outcome on the Emotional Contagion

#### 3.2.1. The Manipulation Checks of Emotional Contagion Presence

Before investigating the effect of reward outcome on emotional contagion, we had to confirm that emotional contagion did in fact occur in this study. Although the occurrence of emotional contagion is typically evaluated by comparing emotional stimuli to neutral stimuli (i.e., less likely to activate emotional contagion), manipulation checks of emotional contagion can also be tested through one-sample *t*-tests for the differences from five (neutral experience) [54]. Thus, the present study employed this approach to assess the occurrence of emotional contagion.

The results indicated that in the before phase, the self-reported experiences exhibited notable distinction from neutral experiences across all conditions (anger-win: *t*_(34)_ = −2.530, *p* = 0.016, Cohen’s d = 0.428; anger-loss: *t*_(34)_ = −5.464, *p* < 0.001, Cohen’s d = 0.924; happy-win: *t*_(34)_ = 10.254, *p* < 0.001, Cohen’s d = 1.733; happy-loss: *t*_(34)_ = 5.896, *p* < 0.001, Cohen’s d = 0.997), indicating successful emotional contagion. In the after phase, on the other hand, only the anger-loss condition and happy-win condition showed the presence of emotional contagion (anger-win: *t*_(34)_ = 1.335, *p* = 0.191, Cohen’s d = 0.226; anger-loss: *t*_(34)_ = −7.125, *p* < 0.001, Cohen’s d = 1.204; happy-win: *t*_(34)_ = 11.667, *p* < 0.001, Cohen’s d = 1.972; happy-loss: *t*_(34)_ = 1.324, *p* = 0.194, Cohen’s d = 0.224), suggesting shifts in the level of emotional contagion in the after phase compared to the before phase.

#### 3.2.2. The Effect of Reward Outcome on the Emotional Contagion

To scrutinize whether and how the monetary game interaction’s reward outcome affected the emotional contagion change (H2), a rmANOVA on the rating scores of participants’ emotional experiences was conducted, with phase (before, after), emotion (anger, happy), and outcome (win, loss) as independent within-subjects variables. To indicate the emotional contagion more clearly, this study utilized the emotional rating score subtracted by 5 (neutral experience) as the subsequent calculation score for emotional contagion.

The results found that the phase × emotion interaction was significant (*F* (1, 34) = 12.364, *p* = 0.001, *η_p_*^2^ = 0.267). A simple effect analysis revealed a significant decrease in emotional contagion under the happy condition (happy: after < before, *p* = 0.004) and the anger condition (anger: after > before, *p* = 0.002) from the before phase to the after phase; additionally, the happy condition elicited a significantly more positive experience than the anger condition in both the before and after phases (before and after: happy > anger, *p*’s < 0.001). The phase × outcome interaction was significant (*F* (1, 34) = 48.736, *p* < 0.001, *η_p_*^2^ = 0.589). Simple effect analysis implied a significant increase in the emotional experience score under the win condition from the before phase to the after phase (win: after > before; *p* < 0.001), while the loss condition displayed a significant decrease in the emotional experience score from the before phase to the after phase (loss: after < before, *p* < 0.001); meanwhile, the win condition evoked a significantly more positive experience than the loss condition in both the before phase and after phase (before and after: win > loss, *p*’s < 0.001). The emotion × outcome interaction yielded a significant result (*F* (1, 34) = 4.968, *p* = 0.033, *η_p_*^2^ = 0.127). Specifically, irrespective of the win or loss condition, the happy condition consistently elicited a more positive state compared to the anger condition (win and loss: happy > anger, *p*’s < 0.001); additionally, the win condition was associated with a more positive experience than the loss condition, regardless of the emotion condition (anger and happy: win > loss, *p*’s < 0.001). The three-way interaction and main effect of phase were not significant. The main effect of emotion was significant (*F* (1, 34) = 44.966, *p* < 0.001, *η_p_*^2^ = 0.569), implying that the contagious emotional experience was significantly more positive in the happy condition compared to the anger condition. The main effect of outcome was also significant (*F* (1, 34) = 55.667, *p* < 0.001, *η_p_*^2^ = 0.621), with the score under the win condition significantly more positive than that under the loss condition. These results revealed that there were significant positive changes in emotional contagion when the reward outcome was a win or the expresser’s emotion was anger. Conversely, the opposite pattern was observed when the outcome was a loss or the expresser’s emotion was happy (see Figure 2C,D).

### 3.3. Control Analysis

To assess the potential interference of rewarding emotions arising from the reward outcomes (such as the pleasure of giving a win) on the present research findings, this study also undertook supplementary analyses pertaining to emotional contagion (as measured by the emotional experience). Specifically, if the reward outcome elicited a strong rewarding emotion (e.g., pleasure for win), we would anticipate a mitigating effect of a win (rewarding pleasure) at trial N on anger (negative emotion) contagion at Trial N + 1 in the before phase. Moreover, the mitigation impact of rewarding pleasure on the anger contagion, in comparison to the happy contagion, would be expected to be more pronounced in the after phase than in the before phase. 

However, this was not the case. The paired *t*-tests on emotional experiences revealed no significant difference between the anger-before win and the anger-before loss conditions in the before phase (*t*_(34)_ = 1.174, *p* = 0.103, Cohen’s d = 0.283), thus denying the mitigating effect of a win (rewarding pleasure) at trial N on anger (negative emotion) contagion at Trial N + 1 in the before phase (as shown in the comparison between anger-before win vs. anger-before loss). In addition, the mitagting impact of a win on anger contagion, in comparison to happy contagion, was not more pronounced in the after phase than in the before phase, as indicated by the opposite pattern in differential emotional experience scores (happy-win minus anger-win within the subject) between the after phase and the before phase (after < before, *t*_(34)_ = −2.841, *p* = 0.008, Cohen’s d = 0.480), indicating that the level of emotional experience was not solely determined by rewarding pleasure (especially under the anger emotion). Taken together, these findings suggested that rewarding emotion alone was insufficient to fully facilitate changes in closeness from the before phase to the after phase. It is plausible that the expressers’ emotion (anger or happy expression) and its transmission (emotional contagion) in the reward context contributed to such changes.

### 3.4. The Effect of Reward Outcome on Brain Activities

**Figure 3:**
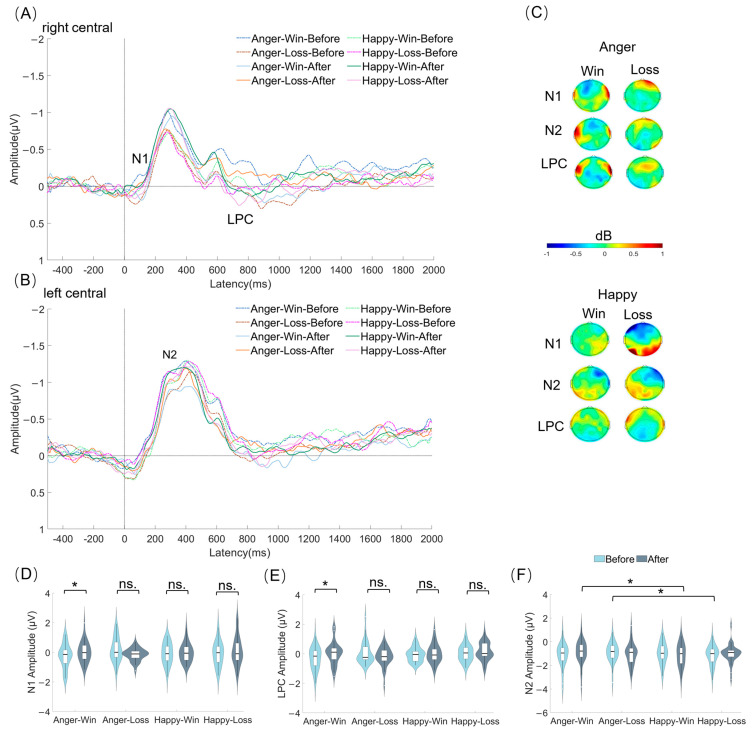
Grand-averaged amplitude of ERPs in the right central area (**A**) and in the left central area (**B**). Topographic map showing the difference between data in the after phase and the before phase (**C**). Statistical histograms of the three components (N1 and LPC: right central; N2: left central) (**D**–**F**). ns.: not significant; *: *p* < 0.05; error bars represent standard errors.

The activity of ERP components (N1, N2, and LPC) in each electrode area were also analyzed by a three-way rmANOVA with the factors of phase (before, after), outcome (win, loss), and emotion (anger, happy) (see Figure 3). The aim of this analysis was to investigate whether changes in ERPs occurred during the experience of others’ emotions following the monetary games, and how the reward outcome influenced these alterations in brain activities (H2).

***N1*.** The phase × outcome interaction was significant in the midline central area (*F* (1, 34) = 6.073, *p* = 0.019, *η_p_*^2^ = 0.152). The simple effect indicated that under the loss condition, the N1 amplitude in the after phase was significantly more negative compared to that in the before phase (loss: after < before, *p* = 0.010), while no significant difference was observed under the win condition (*p* = 0.821). In the before phase, the N1 amplitude was significantly more negative under the win condition compared to that in the loss condition (before: win < loss, *p* = 0.006), but there was no significant difference in the after phase (*p* = 0.856). The phase × emotion interaction also yielded a significant result (right frontal: *F* (1, 34) = 9.438, *p* = 0.004, *η_p_*^2^ = 0.217). The result of simple analysis suggested that under the anger condition, the N1 amplitude showed a marginally significant decrease in negativity from the before phase to the after phase (anger: after > before, *p* = 0.070), whereas no significant distinction was found under the happy condition (*p* = 0.218). In the before phase, the anger condition evoked a more negative N1 amplitude compared to the happy condition (before: anger < happy, *p* < 0.001), yet there was no significant difference in the after phase (*p* = 0.292). The emotion × outcome interaction also yielded a significant result (left central: *F* (1, 34) = 5.103, *p* = 0.030, *η_p_^2^* = 0.130). The results of simple analysis indicated that there was no significant difference between the two outcome conditions under the anger condition (*p* = 0.250). Simultaneously, under the happy condition, the N1 amplitude was significantly more negative under the loss condition compared to that in the win condition (happy: loss < win, *p* = 0.019). In addition, under the loss condition, the happy condition elicited a significantly more negative N1 amplitude compared to that in the anger condition (loss: happy < anger, *p* = 0.014). The three-way interaction of phase × emotion × outcome was significant (right central: *F* (1, 34) = 4.464, *p* = 0.042, *η_p_^2^* = 0.116). Specifically, the anger-win condition displayed a decreased N1 amplitude compared to the anger-loss condition in the after phase (after: anger-win > anger-loss, *p* = 0.017), disrupting the balance observed in the before phase. In addition, in the right central area, the win context led to a reduction in N1 amplitude of the anger emotion from the before phase to the after phase (anger-win: after > before, *p* = 0.044). The phase × emotion interaction and all main effects were not significant.

***N2***. The phase × outcome interaction showed marginal significance in the midline central area (*F* (1, 34) = 4.047, *p* = 0.052, *η_p_^2^* = 0.106). The simple effect analysis revealed that in the before phase, the N2 amplitude under the win condition was significantly more negative than that under the loss condition (before: win < loss; *p* = 0.015), whereas no significant difference was observed in the after phase (*p* = 0.724). The phase × emotion interaction yielded significant results (right frontal: *F* (1, 34) = 9.740, *p* = 0.004, *η_p_*^2^ = 0.223; right central-frontal: *F* (1, 34) = 6.183, *p* = 0.018, *η_p_*^2^ = 0.154; right central: *F* (1, 34) = 6.618, *p* = 0.015, *η_p_*^2^ = 0.163). The simple effect analysis suggested that for the happy emotion, there was a significant increase in the negativity of N2 amplitude from the after phase to the before phase (happy: after < before; right frontal: *p* = 0.001, right central-frontal: *p* = 0.006, right central: *p* = 0.001); in contrast, there was no difference for the anger emotion. The emotion × outcome interaction was significant (right central-frontal: *F* (1, 34) = 7.016, *p* = 0.012, *η_p_*^2^ = 0.171). The simple effect analysis indicated that under the loss condition, the N2 amplitude of the happy emotion was significantly more negative than that of the anger emotion (loss: happy < anger, *p* = 0.012), while no difference was observed under the win condition (*p* = 0.385). Additionally, for the anger emotion, the win condition evoked a more negative N2 amplitude than the loss condition (anger: win < loss, *p* = 0.042), but the opposite pattern was observed for the happy emotion (happy: loss < win, *p* = 0.044). The three-way interaction of phase × emotion × outcome exhibited significance in the left central area (*F* (1, 34) = 5.247, *p* = 0.028, *η_p_*^2^ = 0.134) and marginal significance in the left central-frontal area (*F* (1, 34) = 5.247, *p* = 0.058, *η_p_*^2^ = 0.102). The simple effect analysis revealed that the greater N2 activity (more negative) under the happy-loss condition compared to the anger-loss condition in the before phase (before: happy-loss < anger-loss, left central-frontal: *p* = 0.037, left central: *p* = 0.048) disappeared in the after phase. Moreover, in the win context, the relation between the anger and happy emotions in the before phase was disrupted in the after phase; specifically, the win context led to an increase in N2 activity of the happy emotion compared to that of the anger emotion (after: happy-win < anger-win, left central-frontal: *p* = 0.016, left central: *p* = 0.018). The main effect of phase demonstrated significant results (right frontal: *F* (1, 34) = 5.501, *p* = 0.025, *η_p_*^2^ = 0.139; right central-frontal: *F* (1, 34) = 4.613, *p* = 0.039, *η_p_*^2^ = 0.119), indicating that the N2 amplitude in the after phase was significantly more negative than that in the before phase (after < before). The main effect of emotion was also significant (right frontal: *F* (1, 34) = 5.041, *p* = 0.031, *η_p_*^2^ = 0.129; left central-frontal: *F* (1, 34) = 5.890, *p* = 0.021, *η_p_*^2^ = 0.148), with the N2 amplitude under the happy emotion significantly more negative than that under the anger emotion. There was no significant main effect of outcome.

***LPC***. The phase × outcome interaction was significant in the right central area (*F* (1, 34) = 5.361, *p* = 0.027, *η_p_^2^* = 0.136). The simple effect analysis indicated that the LPC amplitude under the win condition was more positive in the after phase compared to the before phase (win: after > before; *p* = 0.047), while no significant distinction was observed for the loss condition (*p* = 0.563). The three-way interaction of phase × emotion × outcome was significant in the right central area (*F* (1, 34) = 5.262, *p* = 0.028, *η_p_^2^* = 0.134). Further analysis demonstrated that the anger-win condition induced a more positive LPC amplitude in the after phase than in the before phase (anger-win: after > before, *p* = 0.045), suggesting that the LPC amplitude while experiencing other’s anger emotion showed a significant increase under the win condition from the before phase to the after phase. In the before phase, the anger-win condition evoked a LPC amplitude that was less positive compared to both the anger-loss condition (before: anger-loss > anger-win, *p* = 0.048) and the happy-win condition (before: happy-win > anger-win, *p* = 0.008). Whereas in the after phase, the anger-loss elicited a LPC amplitude that was less positive in comparison to both the anger-win condition (after: anger-win > anger-loss, *p* = 0.010) and the happy-loss condition (after: happy-loss > anger-loss, *p* = 0.008). The main effect of emotion showed significant results (midline central: *F* (1, 34) = 5.558, *p* = 0.024, *η_p_^2^* = 0.141; midline central-parietal: *F* (1, 34) = 13.208, *p* = 0.001, *η_p_^2^* = 0.280; right central-parietal: *F* (1, 34) = 5.658, *p* = 0.023, *η_p_^2^* = 0.143; midline parietal: *F* (1, 34) = 14.322, *p* = 0.001, *η_p_^2^* = 0.296), implying that the anger emotion induced a more positive LPC amplitude than the happy emotion. The other main effects and interactions did not reach significance.

### 3.5. Neural Activity Changes Linked to Closeness and Emotional Contagion Changes

To elucidate the underlying neural mechanisms that contributed to the observed shifts in behavior when influenced by the reward outcomes, this study examined the correlation between changes in neural activity (ERPs: N1, N2, LPC) and changes in behavior (closeness and emotional contagion). To simplify matters, the correlation analysis employed the differential data, specifically the data obtained in the after phase minus the data from the before phase of each trial, to demonstrate the observed shifts. 

**Figure 4:**
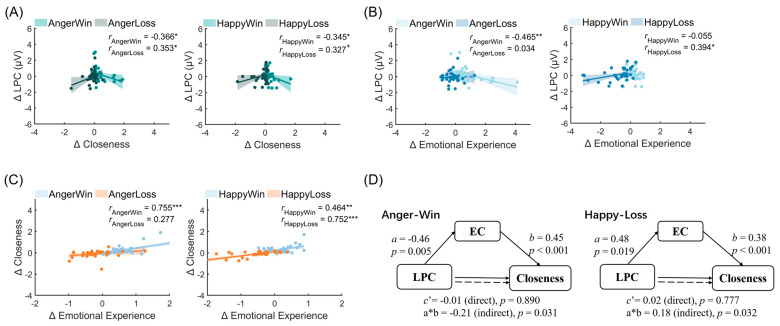
Correlation between changes in closeness and activity in LPC (left central area) (**A**), changes in emotional contagion (EC), and activity in LPC (left central area) (**B**), and changes in closeness and emotional contagion (**C**). (**D**) The decreased change in LPC (left central area) facilitated a positive (less negative) shift in anger contagion, promoting increased intimacy with others. However, it also led to a less pronounced positive change in happy contagion, thereby reducing intimacy with others. ***: *p* < 0.001; **: *p* < 0.01; *: *p* < 0.05; +: *p* < 0.1.

The results revealed that, under the win condition, the amplitude change of LPC (in the left central area) while experiencing others’ anger emotion was significantly negatively correlated with the change in closeness (anger-win: *r*_(34)_ = −0.366, *p* = 0.031), whereas there was a significantly positive correlation under the loss condition (anger-loss: *r*_(34)_ = 0.353, *p* = 0.038). Moreover, the LPC shift associated with the happy emotion was significantly negatively correlated with the closeness shift under the win condition (happy-win: *r*_(34)_ = −0.345, *p* = 0.031), while it showed a marginally significant positive correlation with the closeness change under the loss condition (happy-loss: *r*_(34)_ = 0.327, *p* = 0.055). These results suggested that the increase of intimacy resulting from a giving win in the before phase to the after phase was reflected by a decrease in LPC amplitude; in contrast, the decrease in intimacy generated by a giving loss from the before phase to the after phase was accompanied by the heightened LPC amplitude, as illustrated in Figure 4A.

Additionally, the LPC changes were significantly negatively correlated with the emotional contagion changes with respect to the anger emotion under the win condition (anger-win: *r*_(34)_ = −0.465, *p* = 0.005) rather than under the loss condition (anger-loss: *r*_(34)_ = 0.034, *p* = 0.844). Meanwhile, the LPC changes showed a significant positive association with the shifts in happy contagion under the loss condition (happy-loss: *r*_(34)_ = 0.394, *p* = 0.019); in contrast, no significant correlation was observed under the win condition (happy-win: *r*_(34)_ = −0.055, *p* = 0.753). These findings implied that the anger contagion decreases from the before phase to the after phase (more positive change) due to the impact of a win, possibly indicated by a decrease in LPC activity. Conversely, the diminished happy emotional contagion experienced in the loss context was reflected by a similar trend in the LPC amplitude from the before phase to the after phase, as shown in Figure 4B. Taken together, LPC activity may serve as a neural indicator to track the reward-related change in closeness and emotional contagion.

### 3.6. The Effect of Reward Outcome on the Social Functions of Emotional Contagion

The correlation between emotional contagion change and closeness change before and after the game was examined to explore the relationship between changes in closeness and changes in emotional contagion brought about by reward outcomes (H1 and H3). For simplicity, the correlation analysis also utilized the differential data from each trial to indicate the observed changes. 

The results showed that changes in closeness were significantly positively correlated with changes in happy emotional contagion in both reward contexts (happy-win: *r*_(34)_ = 0.464, *p* = 0.005; happy-loss: *r*_(34)_ = 0.752, *p* < 0.001), suggesting that there was a significant social regulatory function of happy emotional contagion under both outcomes, and the reward outcomes influenced the changes in happy emotional contagion as well as its social functioning in two different aspects. In other words, when the contagious emotion changed more positively, the participants’ closeness toward the expressers became more intimate, whereas when the contagious emotion changed more negatively, the degree of closeness decreased. In addition, closeness shifts were positively associated with shifts of anger contagion, specifically in the win context (anger-win: *r*_(34)_ = 0.755, *p* < 0.001), rather than in the loss context (anger-loss: *r*_(34)_ = 0.277, *p* = 0.108). This finding indicated that winning was more effective in exerting the social function of anger contagion shifts compared to losing, as depicted in Figure 4C.

### 3.7. Mediation Analysis

The abovementioned results revealed that (i) the decreased win-related anger contagion change was associated with a decreased shift in LPC activity (in the left central area); (ii) under all conditions, significant positive correlations were observed between the emotional contagion change and the closeness change; and (iii) the decreased change in LPC activity (in the left central area) indicated a heightened shift in intimacy resulting from the giving win condition. Based on these pairwise relationships and the experimental timeline of each trial in the current study, we inferred that the change in LPC activity (in the left central area) under the anger-win condition may facilitate the subsequent social function of emotional contagion (i.e., the promoting effect of emotional contagion on intimacy with others). 

To verify this conjecture, we used the Multilevel Mediation and Moderation (M3) toolbox in MATLAB [53]. The results demonstrated that the indirect effect of LPC shift (in the win context) on the change in closeness through the change in anger contagion was significant (*b*_ab_ = 0.21, *p* = 0.013; Figure 4D), suggesting that in the win context, the decreased change in LPC activity while experiencing others’ anger emotion facilitated a subsequent less negative shift in anger contagion, which in turn promoted an increased intimacy with others (H4).

In addition, this study conducted similar mediation analyses on data from other conditions, revealing a significant mediating effect not only on the anger-win condition but also on the happy-loss condition (*b*_ab_ = 0.18, *p* = 0.032). Notably, the analysis indicated that the reduced change in LPC activity was found to influence the decrease in intimacy resulting from the giving loss context from the before phase to the after phase, mediated by a less positive change in happy contagion. 

## 4. Discussion

This study aimed to examine the effect of the reward context on the social function of emotional contagion. The results showed that changes in emotional contagion were significantly associated with changes in closeness, thus providing strong support for the social-functional perspective of emotional contagion. In contrast to the context of a loss, the context of a win facilitated a more positive contagious experience for participants, a more intimate closeness toward the expressers, and a larger change in the late cognitive top-down processing of ERP (LPC). Additionally, the change of closeness resulting from the context of reward outcome was reflected by a shift in LPC amplitude from the before phase to the after phase. More importantly, the effect of the reward context on the social regulatory function of emotional contagion reflected duality. It not only demonstrated a positive consequence of emotional contagion but also supplemented the negative aspect of emotional contagion through the reward context. Furthermore, it was found that in the context of a win, the decreased LPC activity while experiencing others’ anger emotion facilitated a subsequent less negative experience rating in contagious anger, which in turn promoted an increased intimacy with others. The results are discussed in detail below. 

### 4.1. The Social Function of Emotional Contagion

The results showed a significant change in closeness after the monetary game interaction (Figure 2B). This indicates that the interaction in which the expresser played games for the participants to obtain reward outcomes could effectively establish connections between the participants and the expressers, changing the participants’ subjective feelings of closeness toward the expressers. This proves the effectiveness of the interactive approach used in this study. Moreover, emotional contagion also underwent changes (Figure 2D) and was found to be positively associated with changes in closeness in both reward and non-reward contexts (Figure 4C). This suggests that changes in emotional contagion have a social regulatory function that promotes changes in closeness (H1). This is consistent with previous studies on the role of emotional contagion in promoting social interaction and relationship quality [4] as well as the social-functional view of contagious responses [2], providing empirical support for the social function of emotional contagion.

Importantly, it was found that in the context of a win, the changes in emotional contagion toward more positive feelings can predict the changes in closeness in the direction of more intimacy, whereas in the context of a loss, the changes in emotional contagion toward more negative feelings predicted the changes in closeness in the direction of estrangement. In line with previous views on the positive consequences of emotional contagion in facilitating closer relationships [4], the current research also expands on the negative consequences of emotional contagion in promoting distance through the reward context. This indicates that the context of rewards affects the relationship between changes in emotional contagion and changes in closeness (H3). 

### 4.2. The Effect of Reward Outcome on the Changes in Closeness, Emotional Contagion, and Brain Activity

Changes in closeness were affected by reward outcomes. Specifically, the win condition promoted changes in closeness in a more intimate direction, whereas the loss condition resulted in changes in closeness in a more distant direction (Figure 2B). This finding is consistent with the expected reward effect, indicating that reward outcome plays a motivational role in promoting changes in interpersonal relationships.

These results revealed that reward outcomes also influenced changes in emotional contagion. The win condition promoted changes in emotional contagion in a more positive direction, whereas the loss condition led to changes in emotional contagion in a more negative direction (Figure 2D). This finding is consistent with previous findings on the contagious response (mimicry). Sim et al. (2012) found that individuals showed greater mimicry of happy emotions in faces associated with a win and insisted that wins promote positive contagious responses [5]. Korb et al. (2019) found similar results [23]; however, they believed that the effect of reward was related to memories associated with loss. The current study examined emotional contagion, which focuses more on internal feelings, and found a comprehensive effect of the two by observing changes in different timelines (the after minus the before phase). This may indicate the overall effect of reward outcome rather than the greater role of a single win or loss. Moreover, previous research on the reward effect has focused on its impact on contagious responses (mimicry), whereas the emergence of contagious responses does not necessarily predict emotional contagion. The two are associated [55] but do not necessarily co-occur [2,56,57]. This study also provides evidence that reward outcome affects the occurrence and changes in emotional contagion.

In addition, brain activities while experiencing the emotions of expressers were found to undergo significant changes after the monetary game. Reward outcomes also modulated these changes. Specifically, for the anger emotion, the activity of LPC changed significantly more positively (from the before phase to the after phase) under the win condition (compared with the loss), while the activity of N1 underwent opposite changes (Figure 3D,E). Previous findings have shown associations between an early automatic component (N1, N2) with emotional arousal and emotional contagion [33,58,59], as well as associations between LPC activity with late cognitive top-down processing [33,34,35,36], respectively. Thus, the results of our study extend the significance of these brain activities, suggesting that they may occur when participants perceive and experience the emotions of others. The loss context promoted early automatic processing and mirror-like activity, whereas the win context enhanced late cognitive top-down processing.

In contrast, no significant differences were observed in the N1 and LPC activity changes while experiencing others’ happy emotions between the before phase and after phase. This indicated that reward outcomes may exert a greater influence on negative emotions as compared to positive emotions in the current study, which is partially consistent with prior findings suggesting that negative emotional contagion may be more affected by external information [60,61]. Nevertheless, this issue still remains controversial [60,61,62]. Furthermore, the initially observed higher N2 activity of the happy emotion compared to that of the anger emotion in the before phase was not maintained in the after phase within the loss context (Figure 3F). Indeed, previous studies have found a relationship between the N2 component and cognitive monitoring, demonstrating increased activity in response to larger conflicts or incongruities [63,64]. It is conceivable that there might be an elevated level of conflict processing for the happy emotion within the context of loss (compared with the anger emotion within the win context) in the before phase, which aligns with the previous conclusions regarding incongruency processing in the brain. Meanwhile, this conflict might be resolved in the after phase, suggesting that the influence of the rewarding process takes on a greater role. Further research is necessary to validate this reversed inference in the field of changes in experiencing others’ emotions by comparing incongruent conditions with congruent and balanced conditions.

Taken together, the closeness, emotional contagion, and LPC activity changed in a similar direction under different reward outcomes (H2). As a reward is a motivational factor [40], its two aspects correspond to two dimensions of the motivational system [65]. Therefore, it can be suggested that the context of a win may arouse the approaching motivation of participants to facilitate positive consequences. In contrast, the avoidance tendency or aversion brought about by the loss context, although it can increase the level of contagious feelings (more negative), also gives rise to negative consequences (e.g., more distance). 

### 4.3. The Effect of Reward Context on the Social Functioning of Emotional Contagion

The above results show that changes in closeness, emotional contagion, and brain activities were all affected by the reward context. As emotional contagion is a facet of emotional communication, it is plausible that the interactive context may affect social bonds by playing a role in emotional contagion. The results support this hypothesis. 

The brain activities generated by the participants reflected processing while experiencing the emotions of the expressers. The results showed that the changes in LPC activity while experiencing others’ emotion, especially observed in anger emotions within the winning context, was found to be associated with subsequent changes in subjective emotional rating (a measure of emotional contagion) (Figure 4B). This is in line with the previous findings that top-down processing, such as perspective-taking and mentalizing, may amplify or dampen the output of contagion (such as the subjective emotional experience of oneself) [43,44]. Specifically, the smaller the change in the cognitive top-down processing of the anger experience induced by the winning context, the greater the cognitive resources that may be reserved for reward processing to help reduce the subsequent output of anger contagion (less negative change in the subjective experiences of oneself). 

Note that the decrease in LPC activity from the before phase to the after phase under the win condition predicted the quality of more intimate social relationships; conversely, the increased changes in LPC activity under the loss condition were related to more distance (Figure 4A). This indicates that changes in closeness affected by the reward context may be related to changes in the cognitive top-down processing and supports the association between emotional processing and closeness changes. This finding echoes previous studies that found that cognitive top-down processing not only modulates the output of contagion but also elicits a regulated response, involving either approach or avoidance behaviors towards the emotional stimulus [43,44]. That is, the decreased change in cognitive top-down processing resulting from the winning context may encourage approach behaviors, fostering greater intimacy with the emotional expresser. In contrast, an increase in top-down processing change may lead to a more distant relationship.

Furthermore, we extend these findings in new insights on the subsequent change in social facilitation of emotional contagion. The mediation results showed that emotional contagion is a core element in emotional communication; the change in LPC activity activated during experiencing others’ anger emotion could bring changes in closeness by influencing a change in emotional contagion (H4). The results also revealed that the outcome influenced the social functioning of happy contagion. Specifically, a reduced change in LPC activity was associated with a decrease in intimacy resulting from the giving loss context from the before phase to the after phase. This effect was mediated by a less positive change in happy contagion. These findings, together with the results observed in the anger-win condition, suggest a potential selective symmetrical effect of the outcome on emotional contagion and its social functioning. Winning appears to promote the contagion and social functioning of anger, which represents an opposite valence, while losing impairs the contagion and social functioning of happiness, which also represents an opposite valence.

Despite prior findings indicating the influence of cognitive top-down processing on the modulation of emotional contagion output and the subsequent emergence of approach or avoidance behavior, there remains a gap in the literature regarding the explicit examination of the predictive relationship between brain activity and subsequent social functioning in the context of emotional contagion. The mediating mechanism found in the current study not only strengthens the relationship between neural responses to experiencing others’ emotions and subjective experience during emotional contagion, under the influence of a reward context, but also further elucidates the neural mechanisms underlying the modulatory effects of the reward context on the social regulatory functions of emotional contagion. 

### 4.4. Limitations and Future Outlooks

Based on the existing issues mentioned above, this study has other limitations that should be considered in future research. First, it only focuses on the impact of reward outcomes in a monetary game. Other factors besides direct reward motivation, such as the ability of the expresser to express emotions, the relationship between the expresser and the observer, and the expresser’s motivation to express certain emotions [11], may be other boundary conditions that affect the social function of emotional contagion. 

Second, the group and cultural differences between the participants and the expressers may limit the generalizability of the research results. The expression faces in this study were all Caucasian, while the participants were all Chinese university students from Asia, which may have influenced emotional recognition and decoding in this study due to cultural factors. The reason for selecting highly distinguishable faces in this study was originally to simulate emotional contagion interactions between unfamiliar groups to provide evidence and inspiration for exploring world harmony and interpersonal coordination. However, this unfamiliarity may have combined with cultural factors related to race. In the future, unfamiliar expressers of the same race should be selected to explore more general situations in which emotional contagion promotes social bonding. 

Finally, the functional manifestations of emotional contagion may not only promote social bonding. Research has found that individuals can mimic the emotions of those who do not look at them even when these individuals do not live together in social groups, indicating that they do not have good reasons to promote social relationships [11,66]. This suggests that there may be other aspects of the social function of emotional contagion that should be considered. As the predictive theory [67,68] suggests, emotional contagion (including contagious response) is aimed at making social interactions more predictable and improving the efficiency of individual survival and interaction. In this case, the role of emotional contagion may depend on factors other than affiliative motivation, such as prediction encoding [67]. However, specific research evidence remains limited. Therefore, it is necessary to study other functions of emotional contagion in the future to supplement the existing research conclusions on the social function of emotional contagion.

## 5. Conclusions

This study explored the effect of reward outcome on emotional contagion to promote changes in closeness by manipulating the context of rewards. It suggests that the reward context can play a role in motivation, affecting changes in closeness and emotional contagion (as well as the brain activities occurring during experiencing expressers’ emotion). In addition, this study provides a potential neural mechanism whereby LPC activity, elicited during experiencing the expressers’ emotions, facilitates closeness through emotional contagion of the anger emotion in the winning context. Overall, this finding provides a better understanding of how various types of motivation, such as desired gains or unwanted losses, influence the social function of emotional contagion. Building upon this understanding, the novel social interaction paradigm (rewarded assistance by others) and its neurobiological mechanisms influencing the social functioning of emotional contagion, as revealed in this study, may serve as an inspiration and provide a neuroscientific basis for future interventions (e.g., tDCS, TMS, and other neuro regulation techniques) aimed at fostering social relationships and human well-being.

## Data Availability

Study data are available on reasonable request to the corresponding author.
